# A Peptide Binding to the β-Site of APP Improves Spatial Memory and Attenuates Aβ Burden in Alzheimer’s Disease Transgenic Mice

**DOI:** 10.1371/journal.pone.0048540

**Published:** 2012-11-01

**Authors:** Shi-gao Yang, Shao-wei Wang, Min Zhao, Ran Zhang, Wei-wei Zhou, Ya-nan Li, Ya-jing Su, He Zhang, Xiao-lin Yu, Rui-tian Liu

**Affiliations:** 1 Tsinghua University School of Medicine, Haidian District, Beijing, China; 2 National Key Laboratory of Biochemical Engineering, Institute of Process Engineering, Chinese Academy of Sciences, Beijing, China; 3 School of Life Sciences, Ningxia University, Yinchuan, China; Massachusetts General Hospital, United States of America

## Abstract

Amyloid precursor protein cleaving enzyme 1 (BACE1), an aspartyl protease, initiates processing of the amyloid precursor protein (APP) into β-amyloid (Aβ); the peptide likely contributes to development of Alzheimer’s disease (AD). BACE1 is an attractive therapeutic target for AD treatment, but it exhibits other physiological activities and has many other substrates besides APP. Thus, inhibition of BACE1 function may cause adverse side effects. Here, we present a peptide, S1, isolated from a peptide library that selectively inhibits BACE1 hydrolytic activity by binding to the β-proteolytic site on APP and Aβ N-terminal. The S1 peptide significantly reduced Aβ levels *in vitro* and *in vivo* and inhibited Aβ cytotoxicity in SH-SY5Y cells. When applied to APPswe/PS1dE9 double transgenic mice by intracerebroventricular injection, S1 significantly improved the spatial memory as determined by the Morris Water Maze, and also attenuated their Aβ burden. These results indicate that the dual-functional peptide S1 may have therapeutic potential for AD by both reducing Aβ generation and inhibiting Aβ cytotoxicity.

## Introduction

Accumulation and aggregation of β-amyloid (Aβ) likely plays a critical role in AD pathogenesis [1], [2]. Inhibition of Aβ production and prevention of Aβ aggregation, and enhancement of Aβ clearance, are appealing strategies to thwart the onset and progression of AD. Aβ is produced by sequential cleavage of Aβ precursor protein (APP) by β-amyloid precursor protein cleaving enzyme 1 (BACE1) and γ-secretase. BACE1 initiates proteolysis of APP at the N terminus of Aβ, forming a large soluble fragment, sAPPβ, and the remaining membrane-bound C terminal fragment (C-99). C-99 is then cleaved by γ-secretase to form either Aβ40 or Aβ42 [3], [4]. Under normal metabolic conditions, most APP can be processed through an alternative non-amyloidogenic pathway [5]. Alpha-secretase initiates proteolysis of APP at the peptide bond between Lys16 and Leu17 of Aβ, producing the soluble sAPPα fragment and the remaining membrane-bound C terminal fragment (C-83). C-83 is then further cleaved by γ-secretase to form the p3 peptide instead of Aβ. To lower Aβ generation, extensive efforts have targeted α, β and γ-secretase [4], [6], [7]. However, γ-secretase also cleaves other substrates including Notch, and therapeutic inhibition of γ-secretase may lead to toxic side effects, due to the impact on the important signaling pathways and other activities [8]. To avoid these side effects, some γ-secretase modulators (GSMs) which selectively lower Aβ42 without interfering with the physiological function of γ-secretase were studied. The results indicate that GSMs may be promising therapeutics for the treatment of AD [9]–[11]. Previous reports demonstrated that BACE1 levels are elevated in postmortem AD brains [12]–[17] and in neurons around amyloid plaques [18]. Moreover, BACE1 levels rise following physiological stress or injury, such as oxidative stress by Aβ, hypoxia [19], and energy inhibition [20]. Furthermore, overexpression of BACE1 in transgenic mice accelerates amyloid pathology and neurodegeneration. BACE1 has therefore become an attractive therapeutic target for AD, and many BACE1 inhibitors were reported and showed potential application in AD treatment [21]–[23]. However, in addition to APP, many substrates, including P-selectin glycoprotein ligand-1 [24], sialyl transferase ST6Gal [25], [26], β-subunits of voltage-gated sodium channels [27], APP-like proteins [28], and the type III isoform of the epidermal growth factor-like factor neuregulin 1 (type III-NRG1) [29] are also targets for BACE1 cleavage. Besides, BACE1 plays a role in myelination in the peripheral and central nervous systems during development, and may have cognitive and synaptic functions independent of APP processing [29]–[31]. Some reports have indicated that down-regulation of BACE1 reduces Aβ loads effectively and BACE1 knockout mice are healthy, fertile and have no histological pathologies [32]–[34]. Other studies reported serious morbid effects, like early death, reduced size, and cognitive deficits in BACE1-knockout animals, which suggest the potential liabilities of BACE1 inhibition [35], [36]. Therefore, inhibition of BACE1 activity may also block physiological processing, thus leading to various side effects [25], [26], [29]. An agent that can bind to the β-cleavage site of APP may inhibit the production of Aβ without the potential adverse effects of BACE1 inhibition. Similar approaches were demonstrated with a monoclonal antibody and protein that bind to the β-cleavage site of APP [37]–[39]. As of yet, only a few β-site-directed antibodies and few peptide have been reported to improve cognitive function and reduce neuropathology *in vivo* [37], [38], [40], [41]. Using phage display, we report on a peptide that inhibits Aβ generation and cytotoxicity, and attenuates memory deficits and decreases Aβ burden in AD transgenic mice, by binding to the β-secretase cleavage site of APP and to the N-terminal of Aβ.

**Figure 1:**
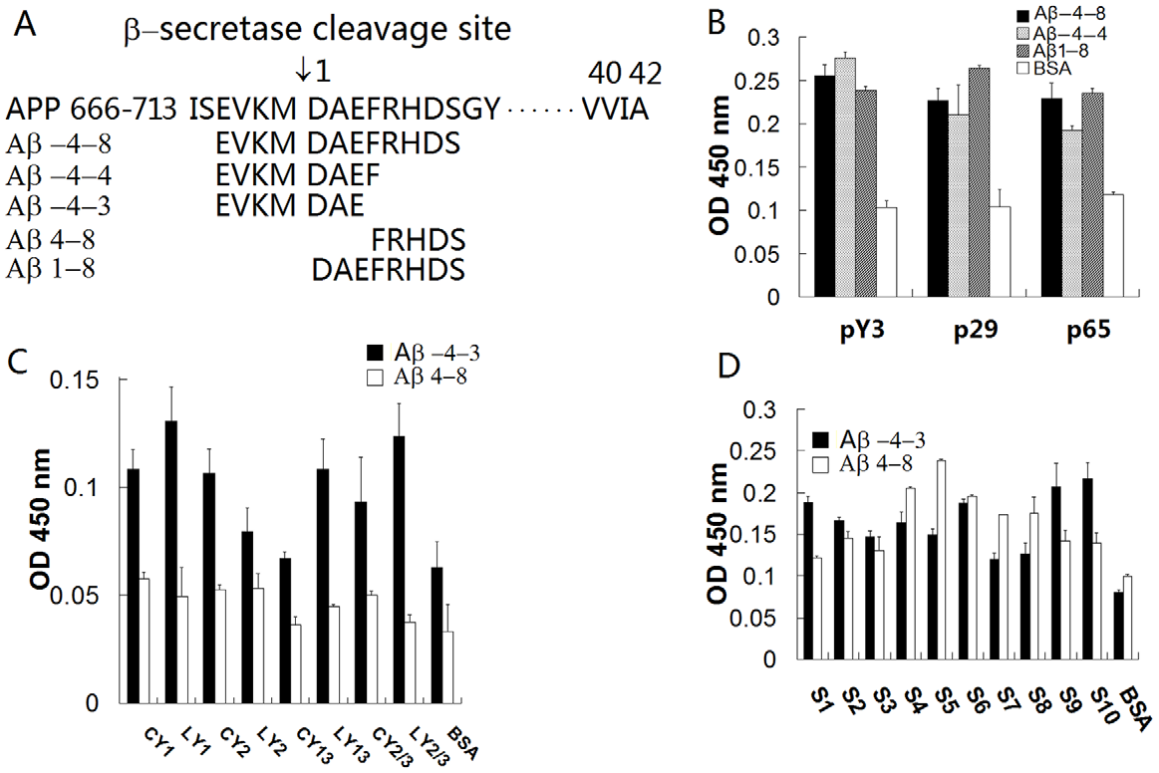
Binding of isolated peptides to the β-secretase cleavage sites of APP and the N-terminal of Aβ. (A) The peptides of the β-secretase cleavage sites of APP and the N-terminal of Aβ used in this study. (B) Aβ-4-8, Aβ-4-4 and Aβ1-8 were coated on an amine-binding 96-well ELISA plate. Three of the selected phage clones were added to the wells. The HRP-9E10 antibody was added to the wells, followed by TMB substrate. Absorbance was read at 450 nm. (C) The peptides displaying positive phage clones were synthesized and coated on an amine-binding 96-well ELISA plate. Aβ-4-3 and Aβ4-8 with His-tags were added to the wells. Mouse anti-His tag antibodies and HRP-conjugated anti-mouse antibodies were sequentially added to the wells and the absorbance was measured as in (B). (D) The chimeric peptides were coated to an amine-binding ELISA plate. Aβ-4-3 and Aβ4-8 with His-tags were added to the wells. Mouse anti-His tag antibodies and HRP-conjugated anti-mouse antibodies were sequentially added to the wells and the absorbance was measured as in (B).

## Materials and Methods

### Materials

The Ph.D.-C7C disulfide constrained peptide library kit encoding 1.2×10^9^ random 7-amino acid insertions was obtained from New England Biolabs. Aβ42 was purchased from the American Peptide Company (Sunnyvale, CA, USA). For Aβ42 preparation, Aβ42 was dissolved in 100% 1,1,1,3,3,3-hexafluoro-2-propanal (HFIP) to a concentration of 1 mg/mL, sonicated in a water bath for 10 min, aliquoted into microcentrifuge tubes, dried under vacuum, and stored at −20°C. Immediately prior to use, the HFIP-treated Aβ42 was dissolved in dimethylsulfoxide (DMSO) to 1 mg/mL and diluted to 10 µM in PBS, pH 7.4. All the other peptides containing the β-secretase site of APP and the N-terminal peptides of Aβ, EVKMDAEFRHDS (Aβ-4-8), EVKMDAEF (Aβ-4-4), EVKMDAE (Aβ-4-3), DAEFRHDS (Aβ1-8) and FRHDS (Aβ4-8) (figure 1A) were synthesized by GL Biochem Ltd. Co (Shanghai, China). The following antibodies were used: A8717 (rabbit polyclonal raised against C-terminal of APP, C99 and C83, Sigma-Aldrich Corp. St. Louis, MO, USA), HRP-9E10 (HRP-conjugated anti-M13 antibody, Santa Cruz, USA), and HRP-conjugated mouse anti-His tag monoclonal antibody (Gold Bridge Co., Beijing, China). Both Aβ40 and Aβ42 kits for Aβ measurement were purchased from IBL Co. Ltd. (Gunma, Japan).

### Bio-Panning Using the Phage Display Library

Peptide selection was performed using three rounds of panning, essentially as described by the manufacturer with slight modifications. Amine-binding 96-well microtiter plates were coated with 1 µg/mL of Aβ-4-8 in PBS (10 mM phosphate, 150 mM NaCl, pH 9.0) overnight at 4°C. Plates were blocked with 5% BSA for at least 2 h at room temperature. An aliquot of 2×10^11^ phage units from the Ph.D.-C7C library was incubated with the peptide. Plates were thoroughly washed with TBST (TBS (50 mM Tris, 150 mM NaCl, pH7.5)+0.1% Tween-20 [v/v]) solution to remove any unbound phage. Bound phage was eluted with 0.2 M glycine-HCl, pH 2.2. Phage titers were determined by infecting Escherichia coli cells and using serial dilutions on agar plates containing IPTG/Xgal. Eluted phages were subsequently amplified by infecting E. coli ER2738. The phage were further purified using polyethylene glycol (MW 8000)/NaCl precipitation, resuspended in PBS and used for further rounds of selection. At the end of round 3 of panning, the eluted phage was diluted, used to infect ER2738 and plated as single colonies on LB plates with IPTG/Xgal. Clones were individually picked, amplified, purified and tested as described in our previous report [42].

**Table 1:** Isolated peptides that bind to Aβ-4-8 by phage display.

Clones	Peptide sequence	Homology
pY3	WQMSPRT	
p29	LQSEPRT	PRT
p2	YSTRQHL	
p18	YWAWQQL	Y×××Q×L
p8	KNPLPGL	
p17	LNSLPYV	N×LP
pY11	LTPFVLT	
p1	GSNTTPF	TPF
pY8	LGMFPPF	
pY12	MSPPWVG	M×PP
pY4	HTHTHPN	
pY10	SHALHNN	S(T)H×H×N
pY12	MSPPWVG	
p65	PQVGAPQ	P×VG
p29	LQSEPRT	
p17	LNSLPYV	L×S×P
p2	YSTRQHL	
p32	LPLHPHL	
pY7	SYAKMHL	HL

**Table 2:** Synthesized peptides.

Original peptide	Sequence	Chimeric peptide	Sequence
LY1	WQMSPRT	S1	PQVGHL
LY2	LQSEPRT	S2	PQVGLQSLP
LY13	PQVGAPQ	S3	PQVGPRT
LY2/3	LQSEPHL	S4	LQSLPQMSP
CY1	CWQMSPRTC	S5	LQSLPHL
CY2	CLQSEPRTC	S6	QMSPPRT
CY13	CPQVGAPQC	S7	QMSPHL
CY2/3	CLQSEPHLC	S8	PRTHL
		S9	PQVGLQSLP QMSP HL
		S10	LQSLPQMSPRT HL

Twenty peptide-binding clones were DNA sequenced and partial peptides corresponding to the encoded sequences were synthesized by standard solid-phase peptide synthesis with N-terminal Fmoc protection. All peptides were purified to homogeneity via reverse-phase high-pressure liquid chromatography and characterized through matrix-assisted laser desorption/ionization-time of flight mass spectrometry [42].

### ELISA

To determinate the affinity of selected peptides to the β-site of APP and the N-terminal of Aβ, amine-binding 96-well microtiter plates were coated with 20 µg/ml of Aβ-4-8, Aβ-4-4 or Aβ1-8 in PBS at 4°C overnight. Non-specific binding was blocked by incubation with 3% (w/v) BSA at 37°C for 1 h. The prepared phages were added to each well and the plates were incubated for 2 h at room temperature. The plates were then incubated with HRP-conjugated anti-M13 antibody followed with TMB substrate. The reaction was stopped after 20 min by addition of 50 µL of 2 N H_2_SO_4_. Absorbance was read at 450 nm on a Tecan Safire2 microplate reader (Tecan, Switzerland).

To further determine the binding of the selected or chimeric peptides to the β-site of APP and the N-terminal of Aβ, amine-binding 96-well microtiter plates were coated with 10 µg/ml of various selected peptides or chimeras. After blocking, the plates were incubated with synthesized His tag-conjugated peptides of Aβ-4-8 and Aβ-4-3 that contain 6 histidine residues (His-tag) at the C-terminus. The plates were then incubated with a mouse anti-His tag monoclonal antibody and HRP-conjugated anti-mouse antibody.

### Thioflavin T (ThT) Fluorescence Assay

ThT dye was used to determine the presence of amyloid-like aggregates. Aβ42 (10 µM) was incubated with or without 20 µM of original or chimeric peptides for 12 or 24 h at 37°C. 20 µl of sample was periodically removed and added to 2 mL of 5 µM ThT solution (50 mM phosphate buffer, pH 6.5). Fluorescence intensity was monitored at an excitation wavelength of 450 nm and an emission wavelength of 482 nm using a Tecan Safire 2 microplate reader (Tecan, Switzerland). All ThT fluorescence experiments were performed in triplicate.

**Figure 2:**
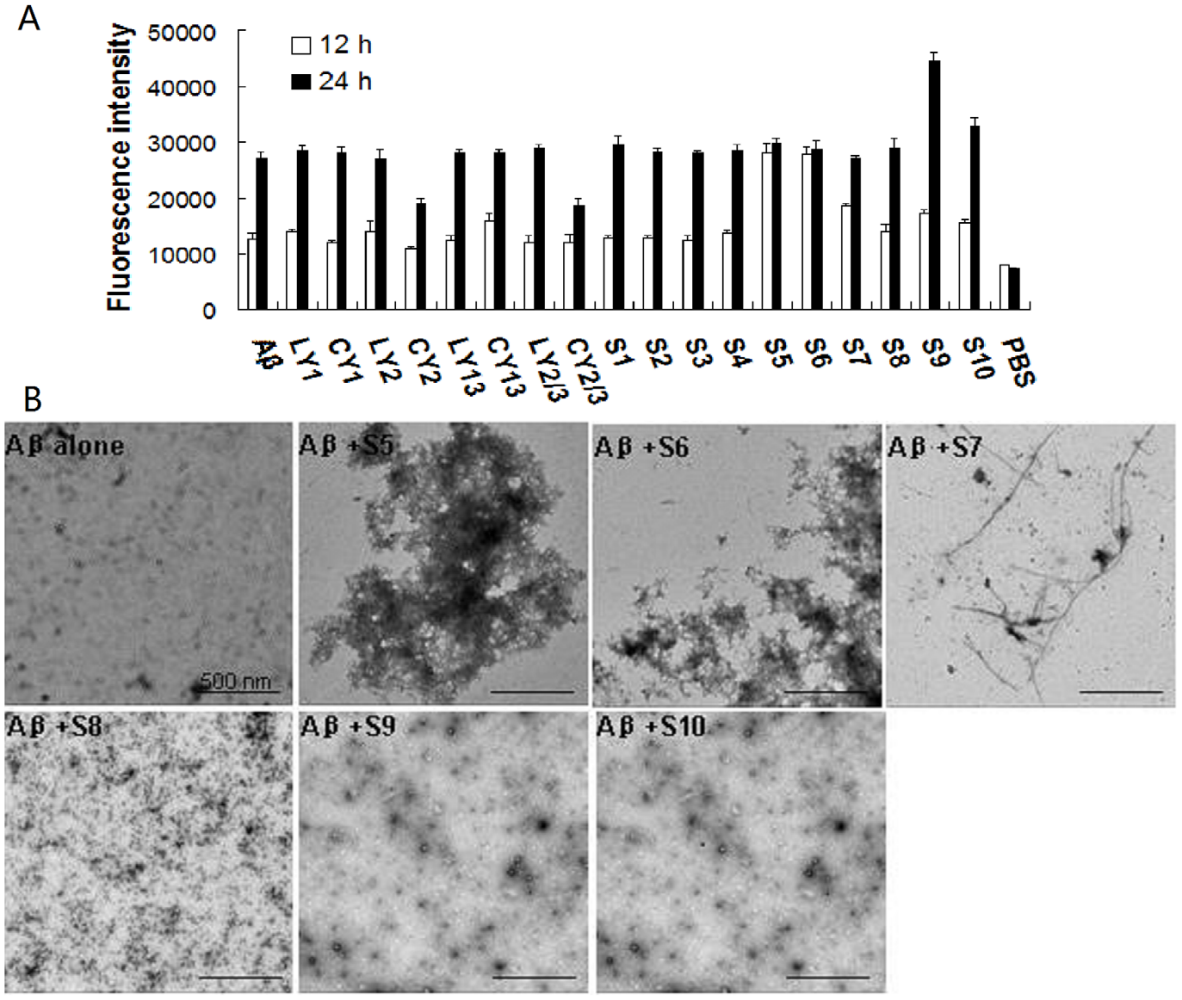
Effect of original and chimeric peptides on Aβ aggregation. (A) Aβ42 (10 µM) was incubated with or without 20 µM of original or chimeric peptides for 12 or 24 h at 37°C. ThT fluorescence intensity was measured with excitation at 450 nm and emission at 482 nm. (B) Ten µl aliquots of Aβ42 incubated with or without peptides S5, S6, S7, S9, and S10 for 12 h were examined with TEM. Images were acquired at 80 kV with 5 K magnification. The scale bar is 500 nm.

### Transmission Electron Microscope (TEM) Imaging

To prepare specimens for TEM imaging, a 10 µL aliquot of each sample was spotted onto a glow-discharged, carbon-coated formvar grid and incubated for 20 min. The droplet then was displaced with an equal volume of 2.5% glutaraldehyde (v/v) and incubated for an additional 5 min. Finally, the grid was stained twice with 10 µl uranyl acetate. The solution was wicked off and then the grid was air-dried. Samples were examined using a Hitachi H7650 transmission electron microscopy (Hitachi, Japan).

**Figure 3:**
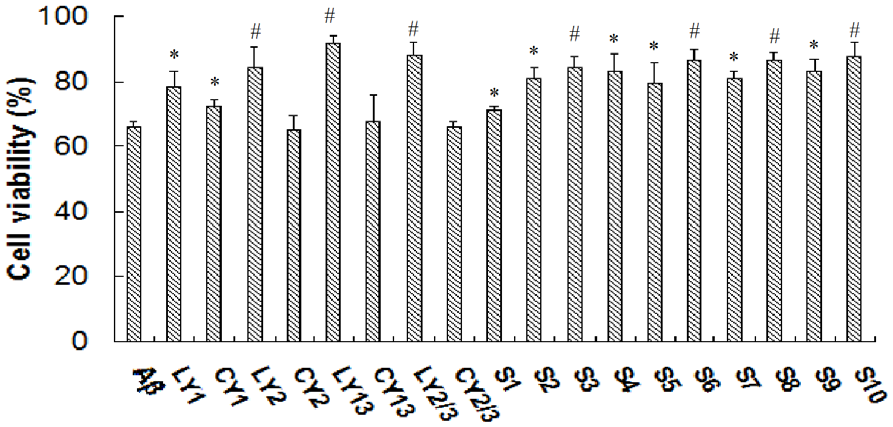
Effect of original and chimeric peptides on Aβ cytotoxicity. Ten µM of Aβ42 with and without original and chimeric peptides was incubated at 37°C for 2 h. The samples were added to wells coated with SH-SY5Y cells with the final concentration of Aβ42 and peptides being 1 µM and 5 µM, respectively. After two additional days of incubation, the viability of the cells was measured using the MTT assay. The absorbance of the dissolved crystals was measured at 560 nm. Data shown were expressed as the percentage of control values (compared with control, *, *P*<0.05; #, *P*<0.01).

### MTT Assay

MTT assay was performed as described previously with light modifications [42]. SH-SY5Y neuroblastoma cells were obtained from the American Type Culture Collection (ATCC) and used to measure cell activity. SH-SY5Y cells were plated in 96-well polystyrene plates with approximately 10,000 cells per 100 µL of medium per well. Plates were incubated at 37°C for 24 h to allow cells to attach. Ten µM of Aβ42 preincubated for 12 h with or without the selected peptide was diluted with fresh medium and added to individual wells with the final concentration of Aβ42 and peptides being 1 µM and 5 µM, respectively. Cell viability was determined with the addition of 10 µL of 5 mg/mL MTT to each well. The plates were incubated for 3 h at 37°C before a 100 µL aliquot of MTT crystal dissolvent (10% SDS and 5% isobutanol in 0.01 N HCl) was added. The absorbance at 560 nm was measured by a Tecan Safire 2 microplate reader (Tecan, Switzerland). Cell viability was calculated by dividing the absorbance of wells containing samples by the absorbance of wells containing medium alone.

### Measurement of Aβ40/42

To determine the effect of the S1 peptide on Aβ production, conditioned media from Chinese hamster ovary (CHO) cells stably transfected to express mutant human APP (Val717Phe; cell line 7PA2 was a generous gift from Dr. Denis Selkoe, Harvard Medical School) were used [43]. For the measurement of intracellular Aβ levels, cells were harvested and extracts were prepared [44]. The amounts of Aβ40 and Aβ42 in the supernatant were detected by sandwich ELISA using Aβ40 and Aβ42 immunoassay kits.

For the measurement of Aβ level in brains, mice were decapitated and brains were removed. One hemisphere (excluding the cerebellum) was homogenized in PBS, pH 7.2, containing a 1× cocktail of protease inhibitors (Calbiochem, Darmstadt, Germany). Brain homogenates were centrifuged at 4°C for 30 min at 15,000 rpm. The supernatant containing soluble Aβ was collected and the pellets were suspended in guanidine buffer (5.0 M guanidine-HCI/50 mM Tris-HCI, pH 8.0). PBS-soluble and insoluble (guanidine soluble) Aβ1-40 and Aβ1-42 were quantified by ELISA according to the manufacturer’s recommendations. The soluble and insoluble Aβ levels were standardized to the brain tissue weight and expressed as ng or mg (Aβ)/g (brain tissue).

### Immunofluorescent Staining

7PA2 cells were fixed with 4% paraformaldehyde for 20 min, perforated with 0.3% Triton X-100 for 10 min, and then blocked with 10% FBS for 60 min, followed by incubation with S1-His tag at 4°C overnight. Cells were incubated with mouse anti-His tag antibody and rabbit anti-APP antibody (Sigma-Aldrich Corp. St. Louis, MO, USA) at 4°C overnight, and then incubated with the secondary antibodies, goat anti-mouse IgG/TRITC and goat anti-rabbit IgG/FITC (Jackson Research Laboratories) for 1 h and counterstained with DAPI for 5 min. Finally, the immunostained cells were visualized with a confocal laser scanning microscope (Olympus BX61).

### Immunoprecipitation

To confirm the binding of S1 to APP, 7PA2 cell lysates were incubated with 200 µM S1-His tag at 4°C overnight. Then the cell lysates were incubated with 1 µg mouse anti-His tag antibody and 30 µl of 50% (v/v) protein G agarose beads (Santa Cruz Biotechnology) for 24 h with gentle rotation at 4°C. The precipitated complexes were fractionated by SDS-PAGE and subjected to Western-blot procedures as below described.

### Western-blot

To detect full-length APP, C99 and C83, the prepared 7PA2 cell lysates or mouse brain extracts were loaded on 16.5% tricine-SDS-PAGE, the separated proteins were transferred to polyvinylidene diﬂuoride (PVDF) membranes (Millipore, USA), the PVDF membranes then were washed and incubated with A8717 (Sigma-Aldrich Corp. St. Louis, MO, USA) for 1 h at room temperature. The membranes were washed again and incubated with the HRP-conjugated anti-rabbit lgG (1∶5000) antibody for 1 h at room temperature. Anti-BACE1 antibody (ab2077, Abcam, Cambridge, UK) was used for detection of BACE-1. The blots were developed with the ECL chemiluminescence kit (Pierce, Rockford, IL, USA), as described by the manufacturer. The intensity of protein bands was quantified using Image J software (National Institutes of Health, USA). Densitometry values are expressed as values relative to β-actin.

To detect sAPPα and sAPPβ, the collected or 40 times concentrated cell culture media were loaded on 10% SDS-PAGE gels, respectively and the separated proteins were detected by Western-blot as described above. The sAPPα and sAPPβ bands were probed with anti-Aβ 3–16 antibody 6E10 and rabbit anti-sAPPβ antibody (9B5211, American Research Products Inc., Palos Verdes Estates, CA, USA), respectively. Densitometry values are expressed as values relative to buffer control samples.

### Animal Treatment

APPswe/PS1dE9 double transgenic mice with a C57BL/6J background were purchased from Jackson Laboratories (Bar Harbor, ME, USA). Genotypes were confirmed by PCR, and in all experiments wild-type littermates served as controls. The transgenic mice and their littermate control (WT) mice (females, 8 months of age) were housed in groups of 9–10 animals, and maintained with access to food and water ad libitum in a colony room kept at 22±2°C and 50±5% humidity, under a 12∶12 h light/dark cycle. All mice were anesthetized with intraperitoneal injection of a combination of 100 mg/kg ketamine and 10 mg/kg xylazine (100 µl/10 g body weight) and were positioned in a stereotaxic apparatus with a mouse head adaptor. The intracerebroventricular (i.c.v) injection was carried out in accordance with the procedure established by Haley and McCormick with minor modifications [45]. An incision was made in the scalp, the skull was exposed and burr holes were drilled in the skull over the injection site. Coordinates for i.c.v. injection were 1.8 mm caudal to bregma, 1.8 mm lateral to midline, 2.5 mm ventral to the brain surface of the skull. Bilateral i.c.v. infusion of selected peptides in PBS (5 µl, 1 mg/ml S1) or PBS (vehicle control) was administrated using a Hamilton microsyringe fitted with a 30-gauge needle. The samples were slowly infused at a rate of 0.2 µl/min. To assure adequate diffusion, the needle was left in place for an additional 5 min before it was slowly retracted. After 7 days, a second i.c.v. injection was carried out. The injections were repeated 4 times. All experimental protocols were approved by the Tsinghua University Animal Care and Use Committee. Experiments were performed according to the code of practice for animal experimentation of the Animal Welfare Act and the Public Health Service Policy on Laboratory Animal Care.

### Morris Water Maze (MWM)

Five days after the last injection, mice were tested for learning and memory abilities in the Morris water maze, as described previously with minor modification [46], [47]. Briefly, mice (9 months of age) were allowed to habituate for 1 week, and then tested in a water maze (1.1 m in diameter) located in a laboratory used for behavioral studies. The water was filled and drained daily. The water was maintained at 22±1°C. The platform (10 cm in diameter) was submerged 1 cm beneath the surface and located at a fixed position (which remained constant throughout the training period), whereas the starting positions were counterbalanced. Swimming activity of each mouse was monitored using a video camera mounted overhead (Sony, Tokyo, Japan), and automatically recorded via a video tracking system. Mice were allowed to swim for 90 s to find the platform where they were allowed to remain for 10 s. Animals unable to locate the platform were guided to it. Mice were trained two times per day over five consecutive days with an inter-trial interval of 3–4 h.

### ThS Staining

Immediately after the behavioral test, mice were decapitated. Brains were rapidly removed and one hemisphere was embedded in tissue freezing medium, snap-frozen in liquid nitrogen and then stored at −80°C. Brains were serially sectioned with a freezing microtome (Leica, Germany) at a thickness of 20 µm and every sixth serial section was selected and mounted onto glass slides, and stained with 1 mg/ml ThS in 70% ethanol. The images were collected on an Olympus BX60 microscope (Olympus Optical Co Ltd, Tokyo, Japan) using a 4× objective.

### Statistics

The data (except behavioral data) presented in this study were obtained from at least three independent experiments for each experimental condition. Data were expressed as means ± SD and their statistical significance was analyzed by one-way ANOVA analysis. Multiple comparisons were performed by Duncan’s test. For evaluation of behavioral data, a two-way repeated-measures ANOVA was utilized to determine statistical differences. All these analyses were performed using SPSS for Windows version 17.0 (SPSS Inc. Chicago, IL, USA).

## Results

### Selection of Peptides

A cysteine-linked phage peptide library (Ph.D.-C7C) was used to identify sequences that targeted Aβ-4-8. Compared with a linear phage peptide in a Ph.D.−7 library, the cysteine-crossed peptides, which have some tertiary structure, may have higher affinities for Aβ-4-8. Twenty positive individual phage clones with the highest phage ELISA values were selected after three rounds of panning. All positive clones were amplified, and the DNA was extracted and sequenced. The level of consensus of sequences was examined using clustalX. Fifteen of the resulting peptides exhibited sequence similarity of three to four amino acids and 3 clones contained the same sequence of two amino acid residues, His-Leu at the C-terminal (table 1). Three positive clones (clones pY3, p29, p65) with a higher affinity for Aβ-4-8 were further confirmed by phage-ELISA. They all bound to Aβ-4-8, Aβ-4-4 and Aβ1-8 (figure 1B). The peptides displayed on these phage clones were synthesized in linear or cysteine-crossed peptide styles (table 2, left column), and the binding to Aβ-4-3 and Aβ4-8 were tested using His tag-conjugated peptides of Aβ-4-3 and Aβ4-8. These linear or cyclic peptides still bound to the β-secretase site of APP, but the affinity was not high (figure 1C). To enhance the affinity to the β-secretase site of APP, some chimeric peptides containing two or more common sequences were synthesized (table 2, right column). His tag-conjugated peptides of Aβ-4-3 and Aβ4-8 were used for the ELISA assay. Peptides S1, S6, S9, and S10, which contained two or three common sequences, exhibited higher affinity to the ligand of Aβ-4-3 (figure 1D), suggesting that the conjugated common sequences may increase the binding to Aβ-4-3. Based on the affinity to Aβ-4-3, the peptides S1, S6, S9, and S10 were selected for further investigation.

### The Effect of the Selected Peptides on Aβ Aggregation

Candidate peptides for AD treatment should not increase Aβ aggregation and cytotoxicity. The effect of the original and chimeric peptides on Aβ42 fibrillation was assayed using the ThT dying assay. When incubated alone, Aβ42 showed the expected increase in fluorescence at the 12 h and 24 h time points (figure 2A). When Aβ42 was co-incubated with S6, its fibrillation was promoted after 12 h while the promoted aggregation was observed in the presence of S9 and S10 after 24 h. Moreover, most original peptides excluding CY2 and CY2/3, and half of the chimeric peptides including S1, S2, S3, S4, and S8, did not show an apparent effect on Aβ42 aggregation (figure 2A).

TEM imaging was used to examine the morphologies of Aβ42 aggregates in the absence or presence of peptides. After 12 h incubation, the sample containing 10 µM Aβ42 alone formed numerous oligomers (figure 2B). However, fibrils were present in the Aβ42 samples incubated with S5, S6, or S7 and numerous protofibrils were present in the Aβ42 samples incubated with S9 and S10, which was consistent with the ThT staining results. After 24 h incubation, all Aβ42 samples in the absence or presence of S9 or S10 formed numerous fibrils (data not shown).

### The Effect of Peptides on Aβ Cytotoxicity

The cytotoxicity of the Aβ42 samples in the presence or absence of synthesized peptides for SH-SY5Y cells was assayed. Cells treated with aliquots of Aβ42 alone showed around a 35% decrease in MTT activity compared to the control wells (figure 3). However, cells treated with the sample of Aβ42 in the presence of S6, S9, and S10 peptides, which promote Aβ42 aggregation, showed an increase of 15–25% in the MTT assay. These findings are consistent with previous reports that the large aggregate morphologies are less toxic to the cells [48]. Although S1 did not show a remarkable effect on Aβ42 aggregation, it could significantly attenuate Aβ42 toxicity. However, CY2 and CY2/3 did not inhibit Aβ42 cytotoxicity, although they inhibited Aβ42 aggregation at 24 h (figure 2A). These results suggest that the aggregation inhibition by CY2 and CY2/3 did not interfere with the toxic aggregate formation. Linear original peptides such as LY1, LY2, LY13, and LY2/3 decreased Aβ42 cytotoxicity, but their cyclic forms (except for CY1) did not have an inhibitory effect on Aβ42 toxicity. Most of the original peptides, linear and cyclic, did not interfere with Aβ42 aggregation. Considering the affinities of peptides to Aβ-4-3, the effects on Aβ42 aggregation and cytotoxicity, S1 was selected for further studies.

**Figure 4:**
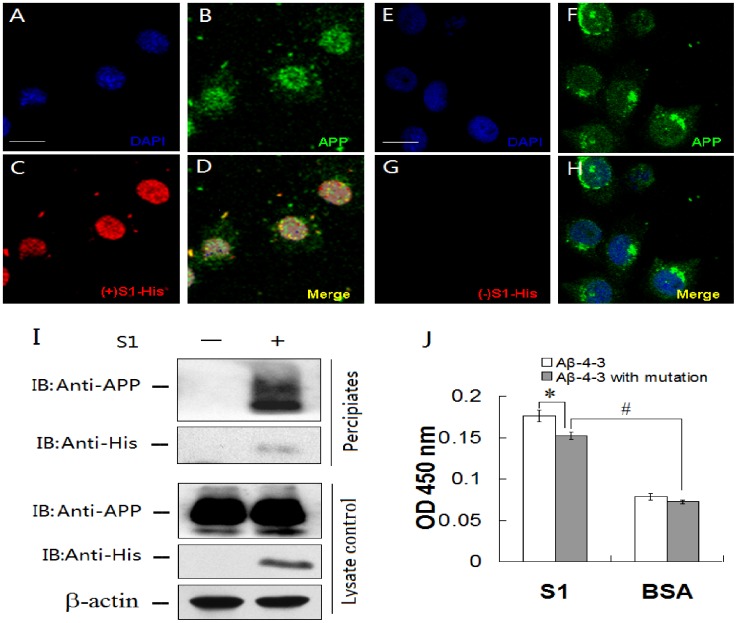
Binding of S1 to cellular APP and mutated β-proteolytic site. 7PA2 cells were incubated with (A–D) or without (E–H) S1-His tag and then stained with DAPI (A, E), anti-APP antibody (B, F) and anti-His tag antibody (C, G), and merged (D, H). The immunostained cells were visualized with a confocal laser scanning microscope (the scale bars = 20 µm). (I) 7PA2 cell lysates were incubated with S1-His tag, anti-His tag antibody and then protein G agarose beads. The precipitated complexes were separated by SDS-PAGE and then detected by Western-blot. Lysates without immunoprecipitation were detected as a control. (J) Binding of S1 to mutated β-proteolytic site was detected by ELISA. S1 was coated to the plate and the muted Aβ -4-3 (M-1A/D-1A) with His tag was added for ELISA assay (*, *P*<0.05; #, *P*<0.01).

### Peptide S1 Binds to Cellular APP

Immunofluorescent staining was used to detect the binding of S1 to APP on/in 7PA2 cells. The cells were stained by anti-APP antibody (figure 4B) and the fluorescence was overlaid by anti-S1-His tag antibody (figure 4C, 4D), indicating that S1 may bind to the APP expressed on/in 7PA2 cells. To further testify these results, cell lysates were precipitated with S1. APP in precipitated complexes was detected using Western-blot (figure 4I).

**Figure 5:**
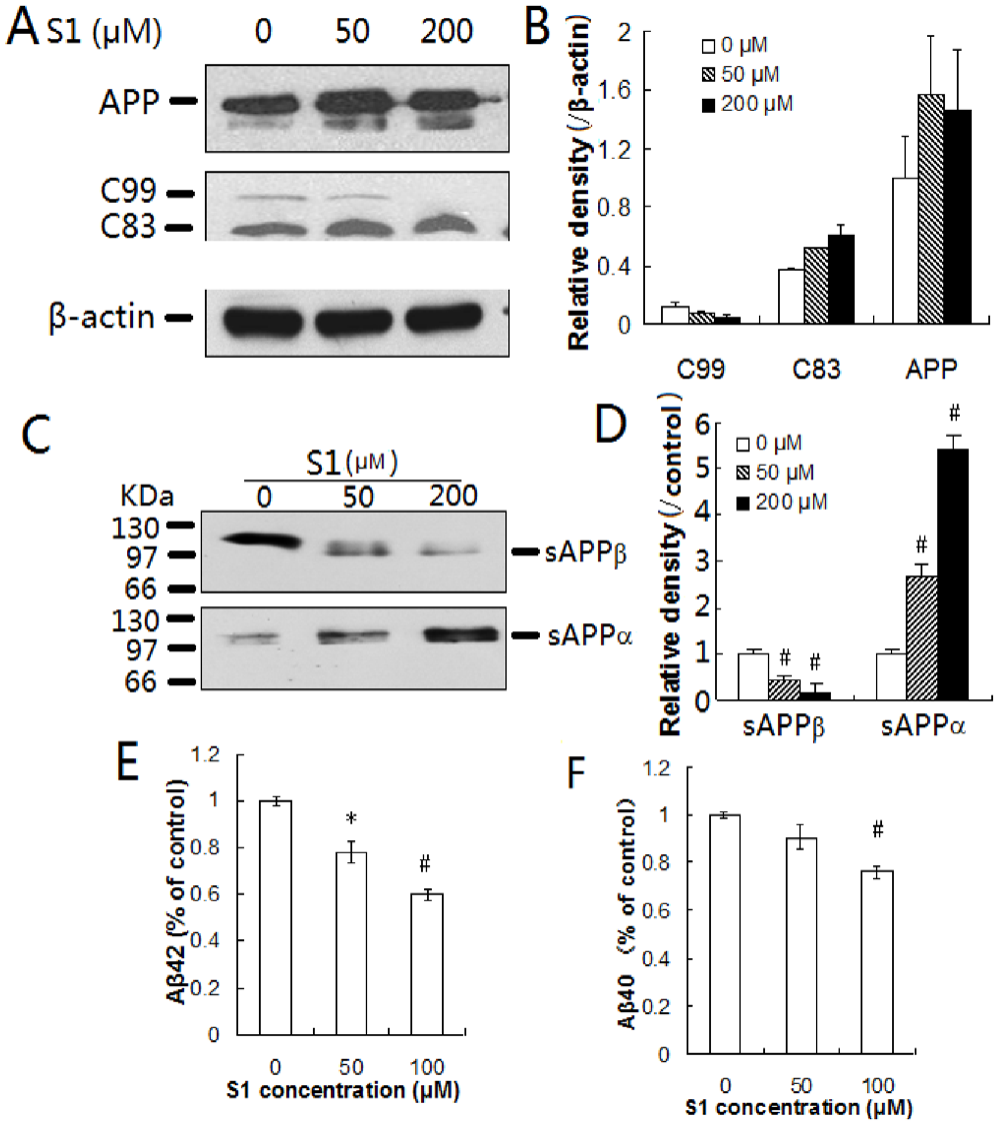
Effect of S1 on APP cleavage and Aβ production in the cells. (A) 7PA2 cells were treated with S1 at 50 and 200 µM for 24 h. Cell lysates were analyzed by Western-blot using antibodies specific for APP, C83, and C99. β-actin in all lysates was used as a loading control. (B) Quantification of APP, C99 and C83 immunoblots in (A) by densitometric analysis. The amount of APP, C99 and C83 in the cells treated with or without S1 is expressed as a value relative to β-actin. (C) sAPPα and sAPPβ in the cell culture media were analyzed by Western-blot using 6E10 and anti-sAPPβ antibody, respectively. (D) The intensity of protein bands in (C) was quantified. Densitometry values are expressed as values relative to buffer control samples. (E–F) Amounts of Aβ40 (E) and Aβ42 (F) in the cell lysates were detected by sandwich ELISA using Aβ40 and Aβ42 immunoassay kits (compared with cell controls, *, *P*<0.05; #, *P*<0.01).

To detect the binding of S1 to muted Aβ -4-3, S1 was coated to the plate and the muted Aβ -4-3 (M-1A/D-1A) with His tag was added for ELISA assay. The OD value of the mutated Aβ -4-3 was significantly decreased compared with Aβ -4-3, but it was still 2 times higher than that of BSA control (figure 4J), indicating that S1 can bind to both beta-proteolytic site of APP and Aβ N-terminal.

**Figure 6:**
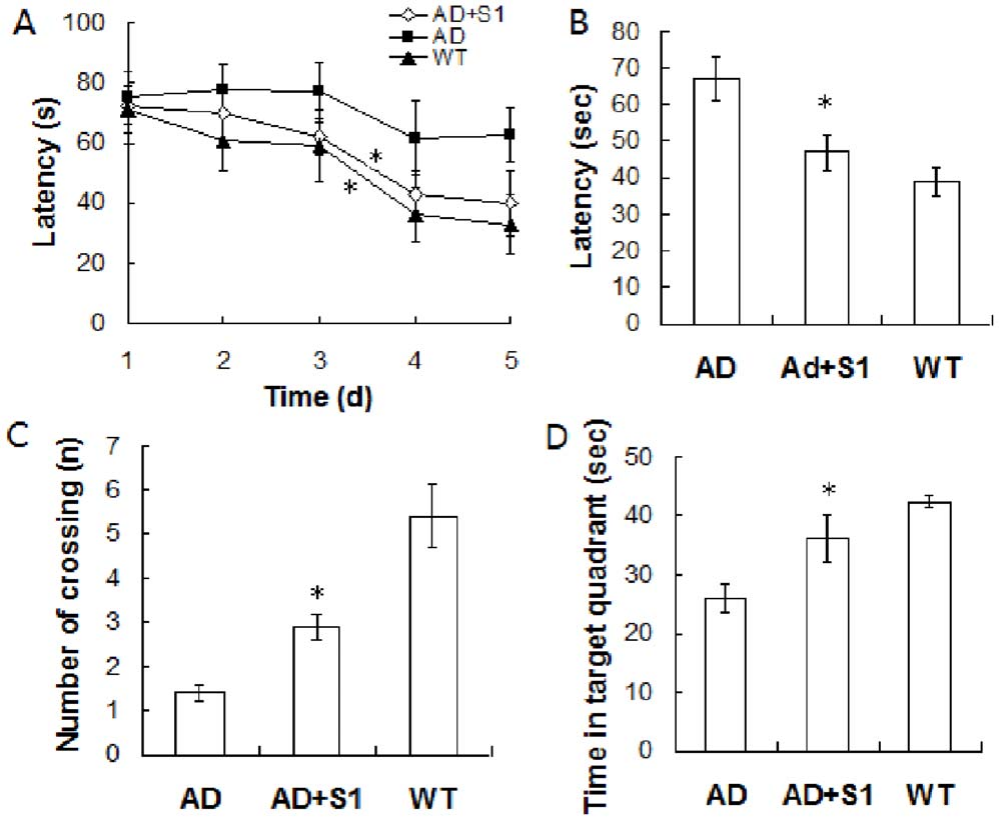
Peptide S1 attenuates spatial memory deficits. (A) APP/PS1 mice were administered S1 (n = 9) or vehicle (n = 9) by i.c.v infusion four times. Littermate control mice (WT, n = 10) were administered vehicle. Each mouse was given two 90 s trials per day for five consecutive days to find the hidden platform in a Morris water maze. The recorded data were analyzed and showed the changes of latency to find the hidden platform over 5 days of the training phase (AD mice treated with S1 or WT mice compared with AD mice control, *, *P*<0.05). (B) Effect of S1 on escape latency during the memory test in the MWM probe trial without a platform. (C) Effect of S1 on the number of crossings (the mice crossed the position where the platform was placed during learning sessions). (D) Effect of S1 on the time spent in the target quadrant during the memory test in the MWM probe trial (compared with AD control mice, *, *P*<0.05).

### Peptide S1 Decreases β-cleavage and Reduces Aβ Production *in vitro*

To investigate the effect of S1 on APP proteolysis and Aβ production, S1 was added to 7PA2 cell culture. Cells were collected and C83, C99 in cell lysates, sAPPβ and sAPPα in cell culture media were analyzed by western blot using the anti-C-terminal of the APP antibody, 6E10 and anti-sAPPβ antibody, respectively. As expected, S1 addition significantly decreased levels of C99 and increased α-secretase–derived C83 in a concentration-dependent manner (figure 5A and B). Consistently, S1 addition significantly decreased levels of sAPPβ and increased sAPPα concentration-dependently (figure 5C and D). S1 addition did not interfere with the levels of APP (figure 5A), indicating that β-cleavage of APP was decreased but α-cleavage was enhanced complementarily.

**Figure 7:**
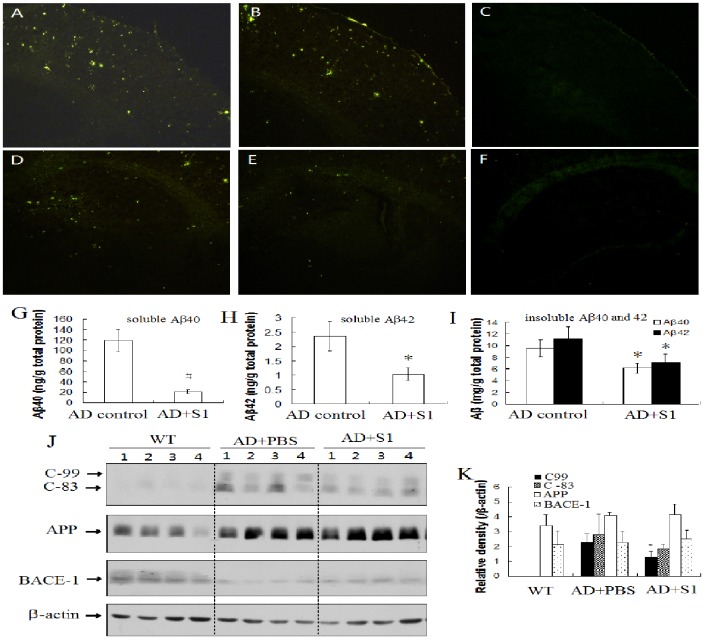
Effect of S1 on amyloid burden and Aβ levels in APP/PS1 transgenic mice. Brain sections were stained with ThS, and the images of the cortex (A, B, C) and hippocampus (D, E, F) of AD transgenic mice treated with vehicle (A, D) or S1 (B, E) and WT mice (C, F) were collected. The amounts of soluble Aβ40 (G), soluble Aβ42 (H) and insoluble Aβ40 and Aβ42 (I) in brain extracts were detected by sandwich ELISA using Aβ40 and Aβ42 immunoassay kits (compared with AD mice control, *, *P*<0.05; #, *P*<0.01). Brain extracts were analyzed by Western-blot using antibodies specific for C83, C99, APP and BACE-1, respectively. β-actin in all extracts was used as a loading control (J). The intensity of C99, C83, APP and BACE-1 immunoblots in (J) was quantified. Densitometry values are expressed as values relative to β-actin (K).

The levels of intracellular Aβ40 and Aβ42 in 7PA2 cells were measured using Aβ40 and Aβ42 immunoassay kits. The S1 peptide at 50 and 100 µM concentrations reduced the amount of intracellular Aβ40 and Aβ42 by 10%, 24%, and 22%, 40%, respectively (figure 5E, F). Both western blot and ELISA results indicated that S1 may significantly decrease Aβ generation.

### Peptide S1 Treatment Attenuates AD-type Cognitive Deterioration

To investigate whether S1 treatment affects learning and memory in AD pathogenesis, behavioral tests were performed using the MWM test after transgenic mice received 5 µg of S1 treatment four times. The wild-type and S1-treated mice exhibited a progressive decrease in escape latency over the 5 day training period (figure 6A). AD mice without S1 treatment also showed improvements in escape latencies after the 5-day training period, but no statistical differences in latencies were observed on each individual day of training, as well as over the 5-day training period. The between-group analysis indicated that S1-treated mice performed significantly better than the vehicle-treated AD mice after 3 days of training. The escape latency of S1-treated mice on the fourth and fifth day of the training test was significantly shorter (43.82±8.30 and 40.56±9.18 s) than that of APP/PS1 mice controls (61.77±11.62 and 62.7±11.35 s; P<0.05; figure 6A). The platform was removed for the probe trial on the day after the last training. Compared with the vehicle-treated AD mice, the WT mice and S1-treated AD mice exhibited spatially oriented swimming behavior. The vehicle-treated AD mice had significantly longer escape latencies (the average time they need to cross the target zone for the first time) than the S1-treated AD mice (figure 6B), suggesting that the latter learned the platform location better. Moreover, the number of platform crossings (calculated by subtracting the average number of crossings over the platform locations in non-target quadrants from the average number of crossings over the exact platform location) by the S1-treated AD mice was significantly higher than that of the vehicle-treated mice (figure 6C). The S1-treated AD mice also spent more time in the target quadrant than the vehicle-treated AD mice (figure 6D). However, the swim speeds of the S1- and vehicle-treated AD mice were equal during the training period and probe trial session (data not shown), indicating that neither group was impaired in motility and exploratory activities. Thus, the S1 treatment significantly improved the memory deficits in APP/PS1 mice.

### Peptide S1 Reduces Amyloid Pathology

To assess the effect of S1 treatment on AD neuropathology, thioflavin S staining was used to detect Aβ-containing neuritic plaques in the brain. As expected, AD mice formed numerous plaques at 9 months of age, but the neuritic plaque formation was slightly decreased in the cortex of S1-treated APP/PS1 mice compared to the controls (figure 7A, B). More interestingly, the plaque burden was noticeably decreased in the hippocampus of mice treated with S1 (figure 7E) relative to the controls (figure 7D).

### Peptide S1 Reduces Aβ Production *in vivo*

To confirm if the amelioration of memory deficits was related to the decrease of Aβ production, all mice were killed after treatment and behavioral tests. The levels of Aβ40 and Aβ42 in the mouse brains were measured. Compared with vehicle-treated APP/PS1 mice control, S1 treatment significantly decreased the levels of both soluble Aβ40 and Aβ42 by 75.2 and 57.6% (figure 7G, H), and insoluble Aβ40 and Aβ42 by 29.6 and 35.8% (figure 7I), respectively, indicating that S1 may inhibit Aβ production *in vivo*.

APP, C83, C99 and BACE1 levels in AD mouse brains were detected using Western-blot. Decreased C99 in AD mice with S1 injection was found, but C83, APP and BACE1 levels did not change noticeably. (Fig. 6J and K).

## Discussion

The cytotoxicity of multimeric aggregates assembled from Aβ monomers has been strongly associated with the neurodegenerative pathology, and the cascade of harmful events related to AD [49], [50]. Therefore, many therapeutic efforts target the inhibition Aβ neurotoxicity or production [42], [44], [51]–[54]. Although numerous inhibitors have been tested to inhibit Aβ aggregation and neurotoxicity, only a small subset of these agents are satisfactory for preclinical drug development. Peptides represent a promising therapeutic approach since they have less adverse effects and have low molecular weights. APP cleavage by BACE1 is the rate-limiting step in the generation of Aβ and plays a key role in the neurodegenerative and behavioral deficits seen in AD [55]–[57]. Therefore, BACE1 is an attractive drug target for the development of new potential drugs. Some BACE1 inhibitors have been developed [58]. These inhibitors include antibodies, peptides and non-peptide compounds that might inhibit Aβ generation *in vitro* and *in vivo* [59]–[62] or ameliorate Aβ pathology and behavioral deficits in a mouse model of AD [63], [64]. However, BACE1 activities may be associated with remyelination, mossy fiber long-term potentiation, and cognitive and emotional functions [30], [32], [33], [65]. Complete prevention of BACE1 may result in certain undesirable side effects [66]. Partial reduction of BACE1 is not sufficient to block the BACE1 elevation during the progression of AD, thus limiting its abilities to reduce cerebral Aβ/C99 levels and rescue memory deficits and cholinergic neurodegeneration [67]. In the cell, BACE1-induced cleavage of APP may occur in the endosomes or other regions, such as on the plasma membrane or in the secretory pathways, resulting in Aβ generation [68].

A more safe and effective therapeutic strategy for AD would be to specifically block BACE1-induced APP cleavage without interfering with BACE1 activity toward other substrates. To accomplish this goal, agents binding to the BACE1 cleavage site in the APP substrate instead of the enzyme are main candidates. To our knowledge, few agents that bound to the β-site of APP and reduced Aβ production *in vitro* has been reported [39]. Here, we present a peptide, S1, which binds to both β-site of APP and Aβ N-terminal, significantly reduces APP cleavage and decreases Aβ production *in vitro* and *in vivo*. S1 may bind to APP on the plasma membrane, or as a small molecule, S1 may efficiently enter cells and bind to APP in recycled endocytic vesicles to inhibit intracellular Aβ production. Besides S1, three other peptides (S6, S9, and S10) also efficiently bound to the β-site of APP and the N-terminal of Aβ (figure 1D), but these 3 peptides increased Aβ42 aggregation (figure 2A and B). Nevertheless, these 3 peptides could inhibit Aβ42 cytotoxicity, which may be because these peptides combined with Aβ and promoted Aβ fibrillation via depletion of monomers or toxic oligomers, reducing the formation or the half-life of oligomers. Consistent with these results, several agents, such as ellagic acid and methylene blue, which increase Aβ aggregation, were reported to inhibit cytotoxicity by promoting fibril formation via promotion of both filament nucleation and elongation [48], [69].

Different domains of Aβ may play different roles in Aβ aggregation and the antibodies against different Aβ domains may exhibit different effects on Aβ aggregation [70], [71]. Also, agents with similar structures may exhibit different effects on Aβ aggregation [54]. The N-terminal domain of Aβ peptides, such as D1-A2, affect Aβ aggregation, although these amino acid residues are unmodified Aβ peptides in defining its secondary structure [72]. Aβ40 formed long fibrillar aggregates while an Aβ variant (D1E/A2V) formed only protofibrillar aggregates under the same *in vitro* incubation conditions [73]. N-terminal deletions enhance the aggregation of β-amyloid into neurotoxic, β-sheet fibrils [74]. Therefore, it is understandable that our selected peptides that bound to the N-terminal of Aβ had different effects on Aβ aggregation. Our results show that the chimeric peptides, S1–S4, did not interfere with Aβ aggregation while the S5–S10 peptides facilitated aggregation (figure 2A and B). A previous report demonstrated that the ability of a peptide to promote aggregation correlated with its affinity for the N-terminal 10 residues of Aβ [75]. However, our results showed that not all peptides, such as S4 and S8, with a higher affinity for Aβ4-8 enhanced aggregation, and peptides S9 and S10 with lower affinity, increased Aβ aggregation. These inconsistent results may be because the N-terminal domain used in the present study contained less than 10 residues of Aβ.

AD is characterized by progressive memory deficits and cognitive impairment. Several reports demonstrate that BACE1-directed compounds used as inhibitors for enzyme activity or expression may ameliorate memory impairment and Aβ pathology in mice [63], [64], [76], [77]. However, there are few reports demonstrating that β-site-directed agents exhibit therapeutic functions in vivo. Our present results show that S1 binds to both β-site of APP and Aβ N-terminal and significantly improves spatial memory in AD transgenic mice. These behavioral improvements were accompanied by reductions in brain Aβ levels and plaque burden (figure 6 and 7). Our results showed that the plaque burden was slightly reduced in the cerebral cortex and was remarkably decreased in the hippocampus. These results may be because the intracerebroventricular injection site is adjacent to the hippocampus to allow easy diffusion of the injected S1 peptide into the hippocampus. As short peptides generally exhibit short half-life in vivo, in order to detect the effect of S1 on the behavior and pathology of AD mice, S1 dose was increased to 5 µg in 5 µl (5 µl of 7.7 mM) per mice. Nevertheless, the Aβ deposits in the cerebral cortex was not apparently reduced. Strangely, C-83 levels in AD mouse with S1 injection did not enhanced complementarily (figure 7J and K). The reason warrants further investigation.

Development of the molecules that block the secretase cleavage sites of APP is a viable approach for the development of drugs for AD that should reduce the potential risk of side effects by the inhibitors of secretases. This study is the first attempt to inhibit both BACE1 activity and Aβ toxicity by blocking the BACE1 cleavage site of APP by means of a site-directed peptide. S1 is a small peptide and may be easily modified to facilitate penetration across the blood-brain barrier (BBB), or to increase the stability in the body. The studies to improve S1 crossing BBB and enhance its half-life *in vivo* should be carried out in the next pre-clinical investigation. Moreover, as a short peptide, S1 is readily available for large-scale synthesis and economical production. Such a dual-functional peptide with minimal side effects is a promising therapeutic approach for AD treatment by inhibiting both Aβ neurotoxicity and generation.
